# Legislation on young people's social media use requires evidence-based decisions

**DOI:** 10.1016/j.eclinm.2026.103816

**Published:** 2026-02-24

**Authors:** eClinicalMedicine

On Dec 10, 2025, Australia implemented its ban on social media for children younger than 16 years, after the Parliament of Australia passed the Online Safety Amendment (Social Media Minimum Age) Act 2024 on Nov 29, 2024. Social media platforms must now introduce age–verification processes to prevent children creating accounts or keeping those they already own. It is believed that the ban will protect the mental health and wellbeing of children and adolescents, after a study commissioned by Australia's government found that 96% of children aged 10–15 years were users of social media, of which approximately 70% had seen harmful content, approximately 14% had experienced grooming behaviour, and more than half had experienced cybervictimisation. The ban currently applies to Facebook, Instagram, Kick, Reddit, Snapchat, Threads, TikTok, Twitch, X, and YouTube, and the companies could receive fines of up to AU$50 million if they do not comply.

Several countries have shown signs that they will be following Australia's lead. The EU's European Commission is studying Australia's ban, and MEPs passed a resolution calling for social media bans in November, 2025. Since then, EU member states, including France, Spain, and Greece, have indicated that they will be pursuing bans. Additionally, the UK Government is considering legislation, with the results of a public consultation due in summer, and opposition parties are pressuring the Government to put children's mental health first and move more quickly to implement a ban. However, despite this growing momentum in favour of social media bans for children, experts are sceptical, and evidence supporting their implementation is weak.

In December 2025, more than 140 academics, experts, and civil society organisations (the Australian Child Rights Taskforce), including paediatricians, psychologists, and media academics, signed an open letter criticising Australia's ban, calling it “too blunt an instrument”. They state that, although the risks of social media to young people are well documented, they should be addressed with an evidence-based response that accounts for the role that digital technologies have in modern childhood. Children use the internet and social media to access information and education, develop social skills, and connect with friends and family, so a blanket ban might not have the positive effects on mental health that legislators hope for. They also point out that the ban cannot be implemented effectively and it may push young people to use alternative sites or methods of accessing social media (eg, VPNs, alternative accounts), which are less easily monitored and more likely to put them at risk. These issues were recently covered in an Editorial in *The Lancet Public Health*. Additionally, UNICEF Australia has criticised the ban stating that it will not address the issues young people face and will prevent them enjoying the benefits of social media. Both groups believe that efforts should instead be directed towards making social media platforms safer for young people.

Indeed, there is room for discussion. A 2022 umbrella review on the effects of social media on adolescent mental health found weak evidence suggesting social media could have both positive and negative effects. Conclusions were found to be largely based on cross-sectional data, so causal relationships could not be inferred. Plus, the research did not address mediators that might explain associations between social media use and mental health, or risk factors that might identify adolescents who are most at risk of negative impacts. A need was therefore identified for longitudinal studies to elucidate the causal direction of associations between mental health and social media use and identify who is most likely to experience the negative impacts and why. A recent longitudinal study found no link between adolescent social media use and mental health, but more is needed.

There is also more nuance to the impact social media might have on mental health through cybervictimisation. A study recently published in *The Lancet Child and Adolescent Health* used a genetically informative longitudinal design, which strengthens causal inference by accounting for alternative explanations, to assess mental health outcomes for adolescents who had experienced cybervictimisation. Adolescents who had experienced cybervictimisation were more likely to report generalised anxiety disorder, major depressive disorder, self-harm or suicide attempt, post-traumatic stress disorder, conduct disorder, and psychotic experiences. However, offline victimisation was found to account moderately or substantially for many of these associations; only the association with generalised anxiety disorder was found to be independent of offline victimisation. These findings suggest that cybervictimisation should be considered within the wider spectrum of victimisation and bullying, and that mental health interventions, including those related to social media, should be considered within this context.

While the effort to protect children from the negative impacts of social media use is a noble one, outright bans ignore the potential of their positive impact, and there is currently still the need for strong evidence supporting their implementation. We lack longitudinal data on the effect of social media on young people's mental health, and there has been no study on how it is affected by limiting access to social media. One such study is underway in the UK (and is independent of the Government's consultation), but more is needed, so that truly informed and evidence-based policy decisions can be made on how best to protect young people in the online world.

